# Ectosomes: A New Mechanism for Non-Exosomal Secretion of Tau Protein

**DOI:** 10.1371/journal.pone.0100760

**Published:** 2014-06-27

**Authors:** Simon Dujardin, Séverine Bégard, Raphaëlle Caillierez, Cédrick Lachaud, Lucie Delattre, Sébastien Carrier, Anne Loyens, Marie-Christine Galas, Luc Bousset, Ronald Melki, Gwennaëlle Aurégan, Philippe Hantraye, Emmanuel Brouillet, Luc Buée, Morvane Colin

**Affiliations:** 1 Inserm, UMR837, Lille, France; 2 Université de Lille, Faculté de Médecine, IMPRT, JPARC, Lille, France; 3 CMRR, CHR, Lille, France; 4 Laboratoire d′Enzymologie et Biochimie Structurales, UPR 3082 CNRS, Gif-sur-Yvette, France; 5 Atomic Energy Commission (CEA), Institute of Biomedical Imaging (I2BM), Molecular Imaging Research Center (MIRCen), Fontenay-aux-Roses, France; 6 CNRS, URA2210, Molecular Imaging Research Center (MIRCen), Fontenay-aux-Roses, France; CSIC/Universidad Autonoma Madrid, Spain

## Abstract

Tau is a microtubule-associated protein that aggregates in neurodegenerative disorders known as tauopathies. Recently, studies have suggested that Tau may be secreted and play a role in neural network signalling. However, once deregulated, secreted Tau may also participate in the spreading of Tau pathology in hierarchical pathways of neurodegeneration. The mechanisms underlying neuron-to-neuron Tau transfer are still unknown; given the known role of extra-cellular vesicles in cell-to-cell communication, we wondered whether these vesicles could carry secreted Tau. We found, among vesicles, that Tau is predominately secreted in ectosomes, which are plasma membrane-originating vesicles, and when it accumulates, the exosomal pathway is activated.

## Introduction

Abundant and abnormal accumulation of the hyperphosphorylated microtubule-associated protein Tau is a pathological feature of the neurodegenerative diseases known as tauopathies [1]. The pathological events leading to neurofibrillary degeneration include reproducible loco-regional patterns of Tau expression that differ among sporadic tauopathies, such as Alzheimer's Disease (AD) [2], [3], Progressive Supranuclear Palsy (PSP) [4] or Argyrophilic Grain Disease (AGD) [5]. This neurodegeneration process has been attributed to a passive release of Tau in the extracellular space during neuronal cell death. There is a growing body of evidence that extracellular forms of Tau could play a major role in the spatiotemporal evolution of the degenerating process [6] and could act on vulnerable neurons in neural circuits through a trans-synaptic mechanism [7], [8]. Neither the function nor the mechanism of Tau release into the interstitial fluid (ISF)/cerebrospinal fluid (CSF) is currently understood. Tau is not only associated with microtubules but also localises in other sub-cellular compartments, such as the nucleus [9] and plasma membrane [10], [11], which suggests that this protein has yet unknown physiological functions. Extracellular localisation of Tau may also imply new physiological functions that are altered during the neurodegeneration process [12]. Recent *in vitro* models have established that Tau can be secreted in physiological [13], [14] or pathological conditions [14]–[20]; however, how Tau is secreted is not yet understood. Some data have indicated the presence of Tau in exosomes [16], [18]; however, Tau also appears to be secreted in a free and non-vesicular form [13], [14]. The mechanism of Tau secretion remains to be elucidated with regard to the pathological process. Although spreading of Tau has been demonstrated in murine models, there is no direct evidence *in vivo* that Tau is secreted. Here, we investigated whether secreted Tau is present in extracellular vesicles in physiological and pathological conditions.

Two main types of extracellular vesicles are defined according to their biogenesis: exosomes and ectosomes [21]. Exosomes are small membranous vesicles (40–100 nm) produced by the endocytosis of molecules. Once internalised, endocytosed molecules are either recycled to the plasma membrane (PM) or trafficked to multivesicular bodies (MVBs). The fusion of MVBs with the PM results in the release of exosomes [22], [23]. Ectosomes are larger extracellular vesicles (50–1000 nm) that directly shed from cells by PM budding [24]–[27].

Tau, a soluble cytoplasmic protein, is not directed to the classical secretory endoplasmic reticulum-Golgi secretory pathway in physiological conditions. The association of Tau with the PM is known from many years [10], [28]–[31] and suggests direct vesicle shedding. In addition, considering that ectosomes are released through cell membrane activation by mediators such as intracellular levels of calcium, inflammatory molecules or oxidative stress, which are involved in the physiopathology of tauopathies [27], [32], [33], ectosomes are good candidates as the mechanism of secreting Tau protein in the physiopathology of tauopathies. In this context, we postulated that ectosomes could drive the secretion of Tau. Using several models (*in vitro* and *in vivo*), we demonstrated here that 1) the secretion of Tau is a new physiological process that is, in part, mediated by ectosomal vesicles and 2) pathological over-accumulation of Tau in cells leads to a slight shift towards Tau secretion by exosomal vesicles.

## Materials and Methods

### Ethics statement

The animals were purchased from Janvier Laboratories and housed in a temperature-controlled room maintained on a 12 h day/night cycle with food and water provided ad libitum. The present experimental research has been performed with the approval of an ethics committee (‘Comité d′éthique en expérimentation animale du Nord Pas-de-Calais'-CEEA 342012), it follows internationally recognized guidelines and all efforts were made to minimize suffering.

### Antibodies

The following antibodies were used: a mouse monoclonal antibody (HT7/Thermo Scientific; 1:2000 for biochemistry) that recognises total Tau (AA159 to 163), a polyclonal rabbit antibody against the C-terminus of Tau (C-Ter) that recognises the last 15 AA [34] (C-Ter, raised in-house, 1:10,000 for biochemistry and 1:1000 for electronic microscopy) and a polyclonal rabbit antibody M19G that recognises the N-terminus of Tau (N-Ter, raised in-house; 1:10,000 for biochemistry) [35].

### Viral vectors

The procedures to produce the lentiviral vectors (LV) batches have been previously described [36].

### Cell Cultures

**1) Primary Embryonic Neuronal Cultures.** Rat primary cortical neurons were prepared from 17-days-old Wistar rat embryos as previously described [37]. Extracellular media were collected from primary cultures at 3, 10 and 15 DIV (days in vitro), and the vesicles were prepared. When indicated, primary cells (10 DIV) were infected with LV vectors encoding either GFP or human 1N4R wild-type Tau (h1N4R). After 48 h, vesicles and cell lysates were retrieved and processed.

**2) Cell culture.** N1-E115 cells were cultured as previously described [38]. To generate a stable cell line (N1E115-h1N4R), N1-E115 cells were seeded on 6-well plates and infected by LV vectors encoding h1N4R. Clonal selection was performed using a limit dilution approach, and the level of h1N4R was determined by western blotting. To purify extracellular vesicles, N1-E115 cells were differentiated at 48 hours; the media were then collected, and the vesicles were prepared.

### Lactate DeHydrogenase (LDH) assay

A LDH assay kit was used to evaluate LDH activity released from cells according to the manufacturer's instructions (Cytotoxicity Detection Kit; Roche Applied Science, Meylan, France). Culture media were collected and centrifuged at 250xg for 10 min. The extracellular (LDH_EC_) and intracellular (LDH_IC_) activities of LDH were estimated (absorbance at 490 nm) to determine the ratio of LDH_EC_/LDH_IC_. Statistical inter-group comparisons were performed using an ANOVA with a Bonferroni post-test.

### Isolation of ectosomes and exosomes

Media were collected and placed on ice, and protease inhibitors were added before centrifuging for 10 min at 2,000 g at 4°C. The supernatant was centrifuged for 45 min at 20,000 g. Pellets corresponding to EcEF were resuspended in RIPA buffer (150 mM NaCl, 1% NP40, 0.5% sodium deoxycholate, 0.1% SDS, and 50 mM Tris HCl; pH = 8.0) for biochemical assays or in 4% paraformaldehyde (diluted in phosphate buffer [0.08 M Na_2_HPO_4_ and 0.02 M NaH_2_PO_4_]) for electron microscopy analyses. The supernatant was centrifuged for 50 min at 100,000 g to generate ExEF. Pellets are processed as described for EcEF or resuspended in imidazole 3 mM, pH 7.4 to purified exosomes on linear sucrose gradient (2.25–0.25 M sucrose in imidazole 3 mM pH 7.4). Sample was centrifuged 18 h at 100,000 g at 4°C and 1 mL fractions collected from the top of the gradient. All the fractions were diluted in a final volume of 9 mL of imidazole 3 mM pH 7.4 and centrifuged once 1 h at 150,000 g at 4°C. The corresponding pellets were then resuspended in LDS buffer (Lithium Dodecyl Sulfate 2X containing 100 mM DTT, Invitrogen) for biochemical assays.

### ELISA

Tau fractions (free forms, ectosomes, exosomes) were obtained after ultracentrifugation of the cultured medium as described above. Tau amounts were determined after different dilutions using the INNOTEST hTau Ag (Fujirebio/Innogenetics, Belgium) that is a sandwich ELISA microplate assay for the quantitative determination of human Tau antigen in fluids. Capture antibody is the AT120 antibody and biotinylated antibodies HT7 and BT2 are detecting antibodies [39], [40]. To allow for comparisons among the three fractions, dilution factors were included to compare final concentrations in a volume dependent manner. Data are given in percentage of total Tau in the medium.

### Electrophoresis and Immunoblotting

For cell lysates analyses, cells were rinced once in PBS, scrapped in RIPA buffer. For vesicles analyses, pellets were directly resuspended in RIPA buffer. Protein concentrations were determined (PIERCE ‘BCA Protein Assay Kit') and samples diluted at 1g/L in LDS containing 50 mM of DTT. 10 µg of proteins were denaturated at 100 °C during 10 min, loaded on 4–12% NuPAGE gels (Invitrogen), and transferred to nitrocellulose. Membranes were blocked in Tris-buffered saline, pH 8, 0.05% Tween 20 with 5% skim milk or bovine serum albumin and incubated with the appropriate primary antibody overnight at 4°C. Membranes were then rinced and further incubated with horseradish peroxidase-labeled secondary antibody (goat anti-rabbit or anti-mouse IgGs, Sigma), and bands were visualized by chemiluminescence (ECL, Amersham Biosciences).

To discriminate between extra- or intra-vesicular Tau, EcEF and ExEF from N1E-115-h1N4R or native N1-E115 were purified as described above and were resuspended in imidazole 3 mM pH 7.4. EcEF and ExEF from native N1-E115 were then incubated with recombinant Tau proteins (h1N4R, 2.7 ng/ml) for 30 minutes at 4°C. All vesicular fractions were then incubated with increasing amount of NaCl to detach extra-vesicular Tau (0M, 0.01M, 0.1M, 0.25M or 0.5M). After centrifugation at 100,000 g, 4°C for 50 minutes, fractions were suspended in RIPA buffer The samples were then diluted in LDS buffer for immunoblotting.

### Push-pull ISF collection

Rats were housed and injected (n = 4, two months old) or not (n = 2) with LV encoding h1N4R as previously described [36], [41]. Five months post-delivery, rats were anesthetised (100 mg/kg ketamine and 10 mg/kg xylazine) and placed in a stereotaxic frame. Push-pull probes (11 mm push-pull guide 24G, 11 mm internal canula 33 gauge; Phymep, Paris, France) were implanted on either side of the brain in the CA1 layer of the hippocampus (coordinates relative to bregma: anteroposterior, −5.3 mm; lateral, +6.2 mm; dorsoventral, −6.5 mm). The probes were perfused with artificial CSF (147 mM NaCl, 2.7 mM KCl, 1.2 mM CaCl_2_ and 0.85 mM MgCl_2_) at a rate of 1 µl/min for 90 min.

### Electron microscopy

Vesicle pellets were resuspended in paraformaldehyde overnight at 4°C. Nickel grids were washed with ethanol 100% and placed on a Formvar film (2% in chloroform). Samples (5 µl) were deposited on a grid and incubated for 20 min at room temperature (RT), washed twice with PBS and fixed in glutaraldehyde (1% in PBS) prior to seven washes in distilled water. After incubation for 5 min at RT in 1% uranyl acetate, pH 7.0, the light-sensitive grids were incubated for 10 min on ice in 5% uranyl acetate, pH 4.0-methylcellulose 2%. The grids were then observed under a transmission electron microscope (Zeiss EM902). When indicated, immunolabelling was performed. The grids were rinsed once in PBS and incubated twice (3 min at RT) in PBS-50 mM glycine before incubation in PBS-1% BSA (Bovine Serum Albumin) for 10 min at RT. Primary antibody diluted in PBS (Phosphate Buffer saline)-1% BSA was applied for 1 hour at RT and revealed using an appropriate secondary antibody diluted in PBS-1% BSA (18 nm gold colloidal goat anti-mouse or anti-rabbit 1:20). After rinsing in PBS, the grids were processed as described above.

## Results and Discussion

### Characterisation of ectosomal and exosomal fractions from primary cells

**Figure 1:**
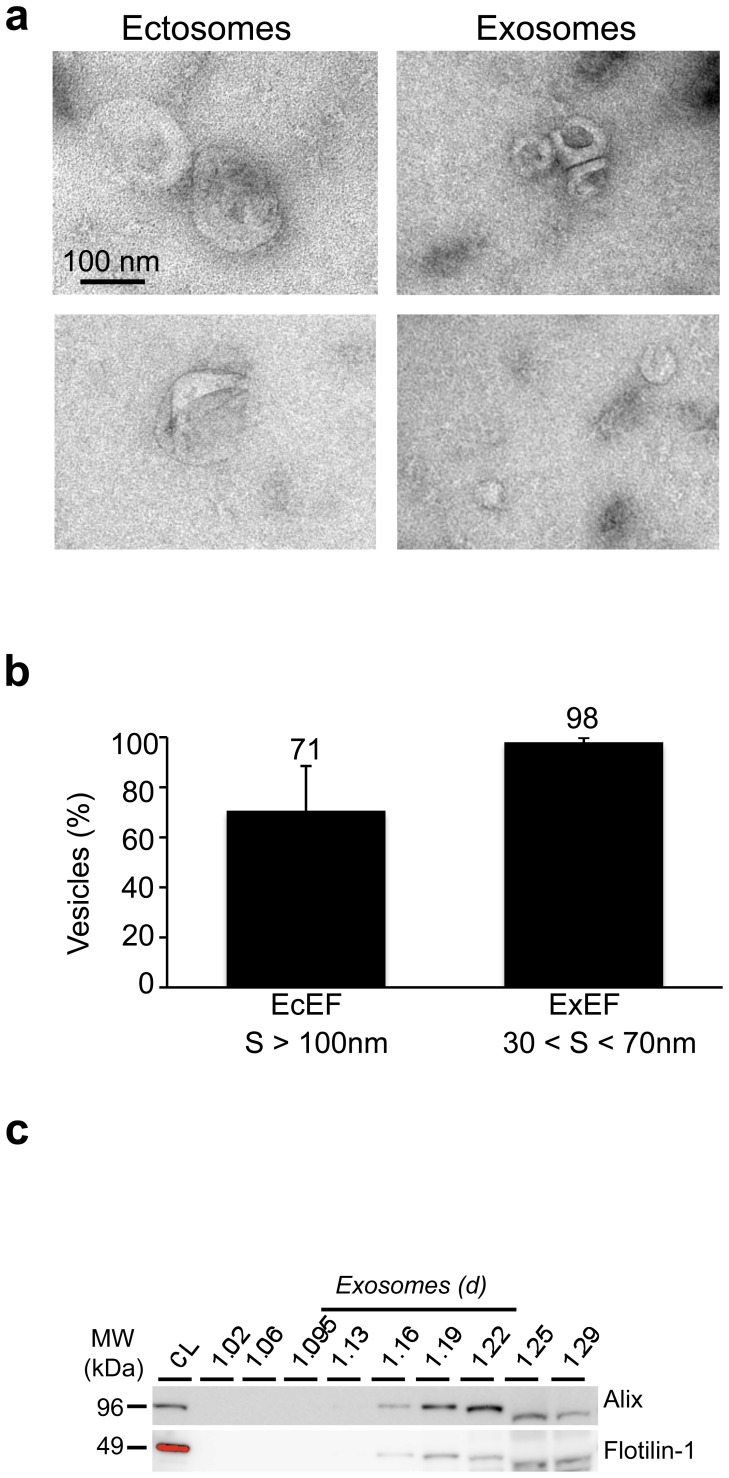
Characterisation of ectomal and exosomal fractions from rat primary embryonic cortical cells. Once purified from embryonic primary cultures, vesicles obtained from EcEF **(a, left panels)** or ExEF **(a, right panels)** were observed by EM. A scale bar is indicated on the figure. **(b)** A semi-quantitative analysis (n = 3 independent experiments with n > 200 vesicles per experiment) was performed to determine the purity of the EcEF and ExEF. The results are expressed as the mean of percentage +/^_^ standard deviation and indicated at the top of the bar chart **(c)** The exosomes were purified from the ExEF using a continous sucrose gradient and Alix and Flotillin-1 used as specific markers.

The ability of primary neuronal cultures to secrete exosomes and ectosomes was analysed. Cell culture supernatants were processed as described in Material and methods section to isolate membrane vesicles. Ectosome-enriched fractions (EcEF, pellet 1) or exosome-enriched fractions (ExEF, pellet 2) were analysed by electron microscopy (EM) (**Fig. 1a**). A semi-quantitative analysis allowed us to determine the purity of these fractions: in EcEF, 71% of vesicles had a size larger than 100 nm, whereas in ExEF, 98% of vesicles had a size between 30 and 70 nm (**Fig. 1b**). Exosomes are vesicles that float on a sucrose gradient and their density ranges from 1.08 to 1.22 g/ml depending their cellular origin [23]. Thus, to further characterize this last pellet, we fractionated ExEF on continuous sucrose gradient and validated the presence of exosomes by biochemical assays using two specific markers, Alix [42] and flotillin-1 [43] (**Fig. 1c**). Thus, in physiological conditions, both ectosomes and exosomes are found in the medium of primary neuronal cell cultures.

### Murine endogenous Tau is physiologically released from rat primary embryonic cortical neurons through a vesicular, but non-exosomal, pathway: the ectosomes

**Figure 2:**
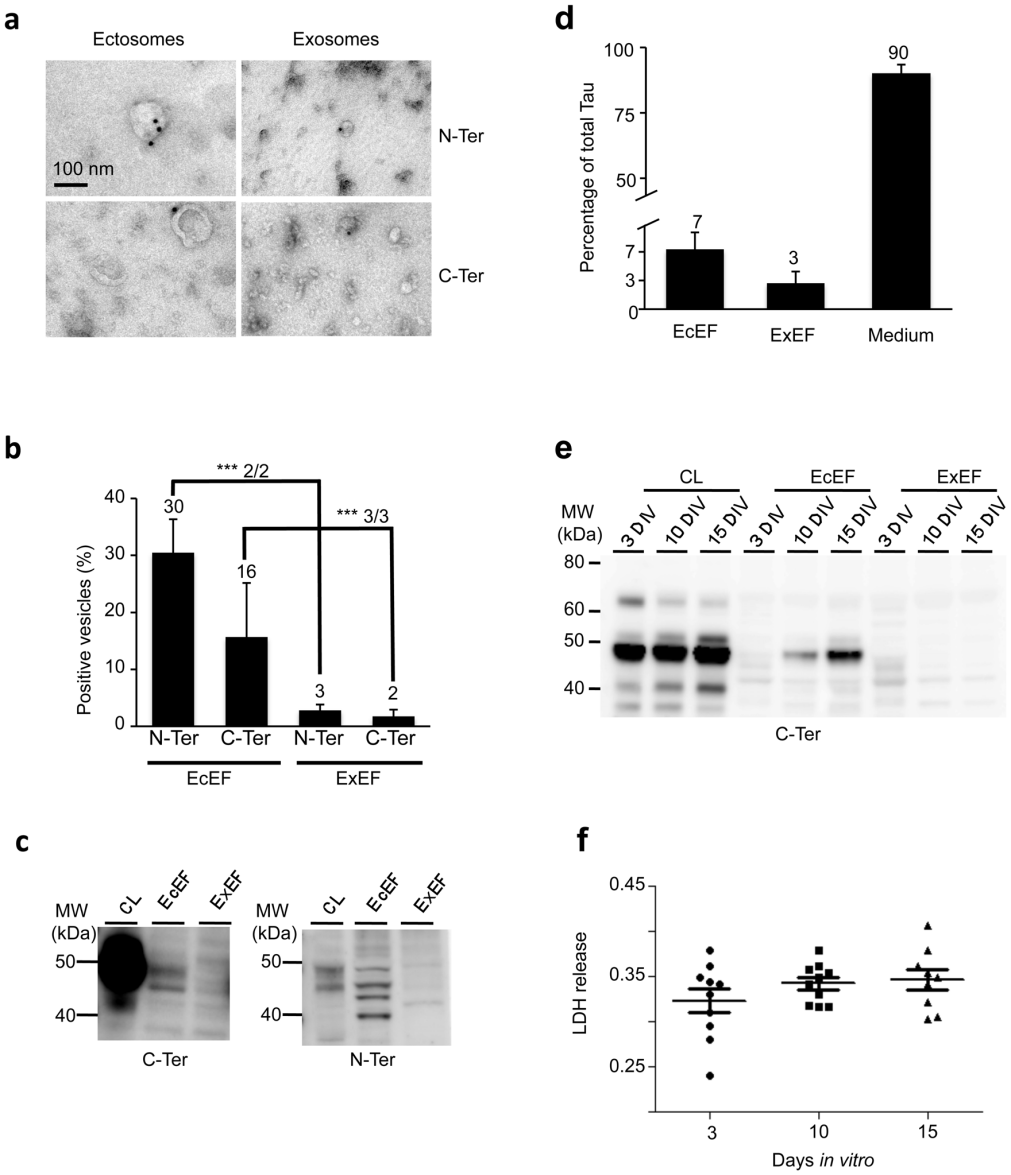
Endogenous Tau is released from rat primary embryonic cortical cells in non-exosomal vesicles: the ectosomes. EcEF (**a, left panels**) and ExEF (**a, right panels**) obtained from primary embryonic cultures were immunolabelled with N-Ter antibodies (**a, upper panels**) or C-Ter antibodies (**a, lower panels**), and the association of Tau with vesicles was observed by EM using an 18 nm gold colloidal goat anti-rabbit antibody. The scale bar is indicated on the figure. **(b)** A semi-quantitative analysis (n = 2 or 3 independent experiments with n > 200 vesicles per experiment) was performed to quantify the percentage of ectosomes and exosomes immunopositive for Tau. The results are expressed as the mean of percentage +/^_^ standard deviation and indicated at the top of the bar chart. The Chi^2^ test was used to compare the presence of Tau in ectosomes and exosomes for each individual experiment: N-Ter: Chi^2^-1 = 100, Chi^2^-2 = 34; C-Ter: Chi^2^-1 = 21, Chi^2^-2 = 20, Chi^2^-3 = 13). The statistical tests indicated p < 0.001 for each condition. **(c)** The presence of endogenous Tau in the cell lysate (CL), EcEF and ExEF obtained from primary embryonic cultures was analysed by western blotting using either C-Ter or N-Ter antibodies. **(d)** A quantitative analysis by ELISA (n = 2 independent experiments) was performed to quantify the ratio of vesicular versus non-vesicular Tau. **(e)** Primary embryonic cultures were plated and maintained in culture for 3, 5 or 10 days (3 DIV, 5 DIV or 10 DIV). Total Tau was analysed by western blotting using the C-Ter antibody in the cell lysate (CL) or after fractionation (EcEF and ExEF) of conditioned media; the conditioned media were also analysed for LDH release (**f**).

The presence of endogenous Tau in ectosome and exosome vesicles was analysed in physiological conditions. Purified vesicles from conditioned media obtained from E17 rat cortical neurons cultured for 10 days (10 DIV) were analysed by electron microscopy. Immunogold labelling of EcEF and ExEF pelleted from these media using antibodies directed against the C- (C-Ter) or the N-terminal (N-Ter) parts of Tau showed a direct association with ectosomes and a poor association with exosomes (**Fig. 2a**). Whereas only 2–3% of exosomes (30 to 70 nm) were Tau-immunoreactive, Tau was associated with 30% (N-Ter) and/or 16% (C-Ter) of ectosomes (larger than 100 nm) (**Fig. 2b**). The biochemical nature of Tau species associated with these vesicles was determined by western blotting. EcEF and ExEF pelleted from the media were processed to reveal the presence of total Tau using either N- or C-Ter antibodies. Tau was predominately associated with EcEF, either in a full-length (50 kDa) or proteolysed forms (**Fig. 2c**). We thus quantified Tau and its proteolytic products by ELISA using antibodies directed against the mid-part of the protein. Tau distribution, in the different fractions: free forms, ectosomes and exosomes, has been estimated to 90%, 7% and 3% respectively (**Fig. 2d**). However, the presence of Tau in the extracellular medium may reflect either active secretion or neuronal cell death. To exclude Tau release by massive cell death, we controlled cell damage induced by neuronal differentiation in our culture conditions. Media from 3, 10 and 15 DIV were collected, and the activity of the cytoplasmic enzyme, LDH was quantified. EcEF and ExEF were then pelleted from these media, and the presence of total Tau was analysed by western blotting. Whereas Tau was progressively secreted in EcEF from the extracellular medium with differentiation (**Fig. 2e**), no change in LDH activity (and thus release) was noted, regardless of the differentiation process (**Fig. 2f**). Thus, Tau secretion occurs in the absence of cell damage. It should be noted that the increase in Tau concentration during neuronal differentiation did not shift Tau secretion from ectosomes to exosomes.

From these results, we concluded that under physiological conditions in rat primary embryonic cortical neurons, Tau is found extracellularly in free forms and actively secreted through vesicular pathways, preferentially in ectosomes rather than in exosomes. Such secreted forms are either in a full-length form or in a C- or N-truncated form. Our data confirm previously published data [12], [13] but also highlight a new mechanism of secretion for physiological release of Tau: membrane plasma-originating ectosomes.

### Characterisation of ectosomal and exosomal fractions from cell lines

**Figure 3:**
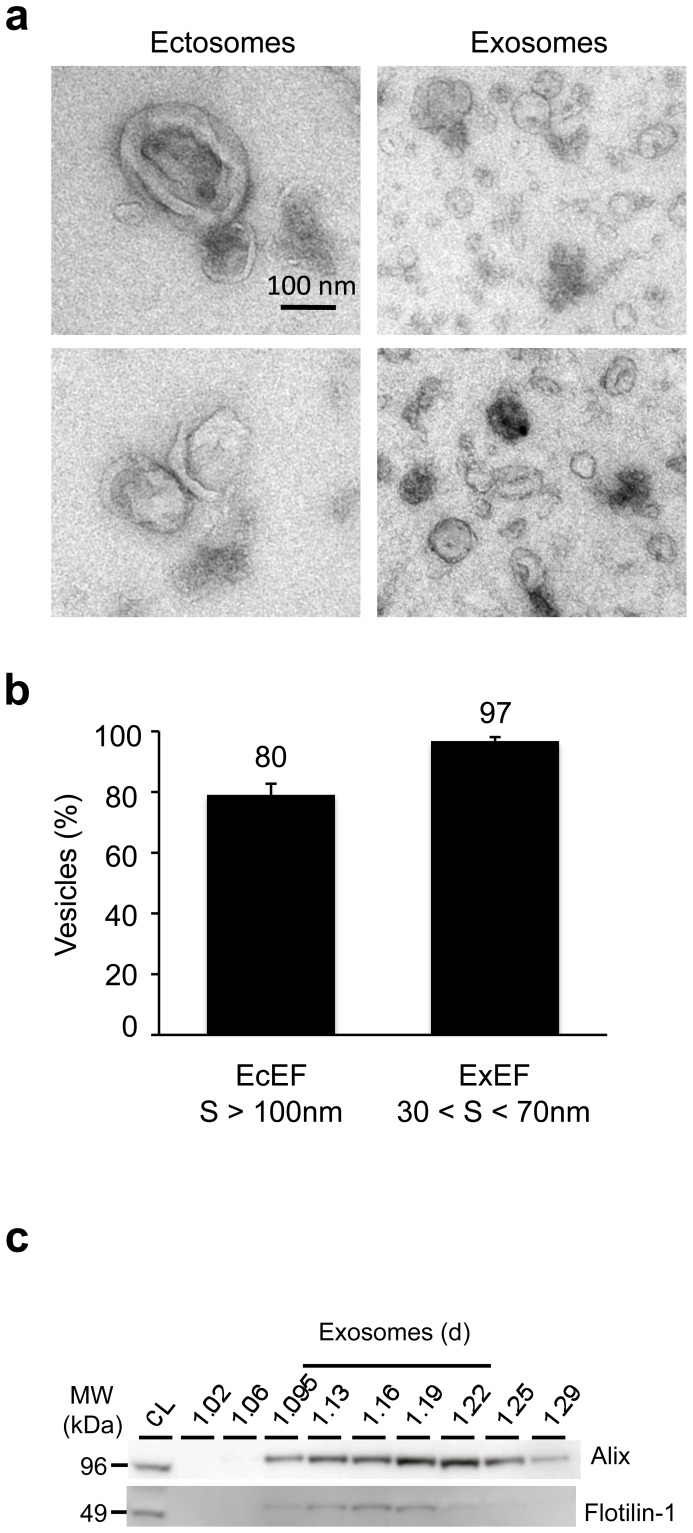
Characterisation of ectosomal and exosomal fractions from cell lines. After purification from N1E-115 cells stably overexpressing h1N4R, vesicles obtained from EcEF **(a, left panels)** or ExEF **(a, right panels)** were observed by EM. The scale bar is indicated on the figure. **(b)** A semi-quantitative analysis (n = 3 independent experiments with n > 200 vesicles per experiment) was performed to determine the purity of EcEF and ExEF. The results are expressed as the mean of percentage +/^_^ standard deviation and indicated at the top of the bar chart. S = size. **(c)** The exosomes were purified from the ExEF using a continous sucrose gradient and Alix and Flotillin-1 used as specific markers.

As previously described for embryonic primary cultures, the ability of neuroblastoma cell lines N1-E115 overexpressing human Tau (h1N4R, 2+3-10+) to secrete exosomes and ectosomes was analysed. EcEF and ExEF pelleted from conditioned media were analysed by EM (**Fig. 3a**). In EcEF, 80% of vesicles had a size larger than 100 nm, whereas in ExEF, 97% of vesicles had a size between 30 and 70 nm (**Fig. 3b**). The presence of exosomes in a continuous gradient was also confirmed by biochemical detection of Alix and flotillin-1 (**Fig. 3c**). As observed using primary neuronal cell cultures, both ectosomes and exosomes are found in the medium of neuroblastoma cells.

### A small amount of Tau is secreted by the classical secretory pathway when Tau over-accumulates in cells

The mechanism supporting cell-to-cell Tau transfer *in vivo* is not yet understood; however, several studies have identified exosomes isolated from cell lines as potential transfer vehicles [16], [18]. One feature in tauopathies is the abnormal accumulation of Tau in neurons. In this context of protein over-accumulation, cells may activate different degradative cellular processes, such as the proteasome pathway and autophagy (for review[44]). For example, the macroautophagy pathway enables the degradation of proteins into lysosomal vesicles through the formation of multivesicular bodies (MVBs). Two distinct populations of MVBs co-exist in cells: the first population targets proteins to lysosomes, and the second population, a cholesterol-rich population, does not fuse with lysosomes but rather drives exosomes outside the cells [45]. Leakage from MVBs could then shuttle Tau outside the cells in exosomal vesicles.

**Figure 4:**
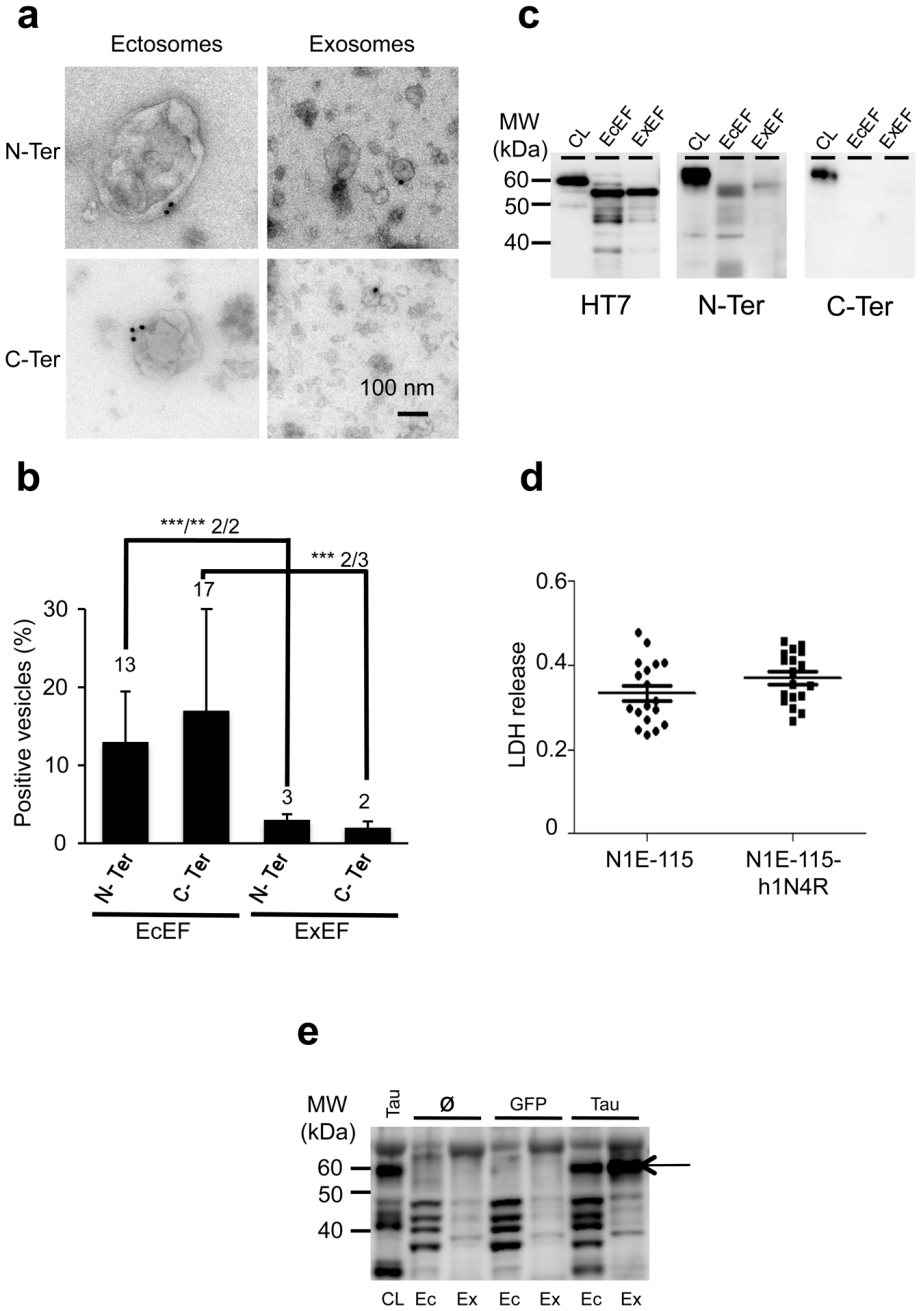
A small portion of Tau is shifted to the classical secretory pathway when Tau over-accumulates in cells. EcEF **(a, left panels)** and ExEF **(a, right panels)** obtained from stable N1E-115 overexpressing h1N4R cells were immunolabelled with N-Ter (**a, upper panels**) or C-Ter (**a, lower panels**) antibodies. The scale bar is indicated on the figure. The association of Tau to vesicles was observed by EM using an 18 nm gold colloidal goat anti-rabbit antibody. **(b)** A semi-quantitative analysis (n = 2 or 3 independent experiments with n > 200 vesicles per experiment) was performed to determine the percentage of ectosomes and exosomes immunopositive for Tau. The Chi^2^ test was used to compare the presence of Tau in ectosomes and exosomes for each individual experiment: N-Ter: Chi^2^-1 = 29 (***, p < 0.001), Chi^2^-2 = 8 (**, p < 0.01), C-Ter: Chi^2^-1 = 22 (***, p < 0.001), Chi^2^-2 = 21 (p < 0.001), Chi^2^-3 = 2.5 (NS)). The results are expressed as the mean +/^_^ standard deviation and indicated at the top of the bar chart. **(c)** Total Tau was analysed by western blotting using HT7, N-Ter and C-Ter antibodies in the cell lysate (CL) or after fractionation (EcEF and ExEF) of conditioned media. **(d)** The conditioned media obtained from native or differentiated N1-E115 cells over-accumulating h1N4R were analysed for LDH release. (**e**) Rat embryonic primary neurons (10 DIV) were infected or not (∅) by LVs encoding either GFP or h1N4R. Total Tau was analysed by western blotting using a N-Ter antibody in the cell lysate (CL) or after fractionation (EcEF and ExEF) of conditioned media.

Therefore, we examined whether this trafficking pathway was involved in Tau secretion in pathological conditions where Tau accumulates in neurons. To test this hypothesis, we generated stable cell lines over-expressing the full-length 4R human Tau isoform (2+3-10+, h1N4R) from N1E-115 cells using lentiviral (LV) technology. Cells were maintained in serum-free conditions to drive differentiation. After 48 h, extracellular vesicles were analysed by electron microscopy as described above to detect Tau in EcEF and ExEF. As observed in primary culture cells (**Fig. 2**), human exogenous Tau was associated with ectosomes (30 to 70 nm) and exosomes (larger than 100 nm) (**Fig. 4a and b**) in the absence of cellular damage (**Fig. 4d**). By immunoblotting, three antibodies were used to determine the nature of the Tau species present in these vesicles: HT7 is a human-specific anti-Tau (epitope within AA 159-163) antibody and the two other antibodies are directed against the N or the C-terminal parts of Tau. EcEF and ExEF are immunopositive for HT7 and N-Ter (**Fig. 4c**). Vesicular Tau species were mainly found in proteolysed forms, in contrast to the cell lysate where the full-length form of Tau was detected (60 kDa). However, in contrast to primary cultures, by immunoblotting, we also found Tau in ExEF from N1E-115 cells stably overexpressing h1N4R. Moreover, the lack of Tau immunoreactivity with the C-Ter antibody in both EcEF and ExEF strongly suggests that in cells over-accumulating Tau, proteolytic fragments lacking the carboxy-terminus are the predominant vesicular forms (**Fig. 4c**). To confirm that Tau accumulation leads to activation of the classical exosomal pathway, h1N4R was over-expressed in rat primary embryonic cortical neurons by lentiviral technology as described for N1-E115 cells (**Fig. 4e**). Primary cells were infected at 10 DIV. After 48 h, cell lysates or Ec/ExEF purified from media were analysed by western blotting using a N-Ter antibody. To control for artefacts arising from the lentiviral technology, primary cells were also transduced with a LV encoding a green fluorescent protein (GFP). When Tau over-accumulated in primary cells, it was strongly detected in both EcEF and ExEF (**Fig. 4e** **, black arrow**). This pattern was not detected after GFP was over-expressed in primary cells or when endogenous Tau was analysed. These results are consistent with those obtained in N1E-115-h1N4R; Tau is also released in the extracellular media using exosomal shuttle vesicles. These findings may explain previously published results in over-expression models [16], [18] where secreted Tau was first described in exosomes.

**Figure 5:**
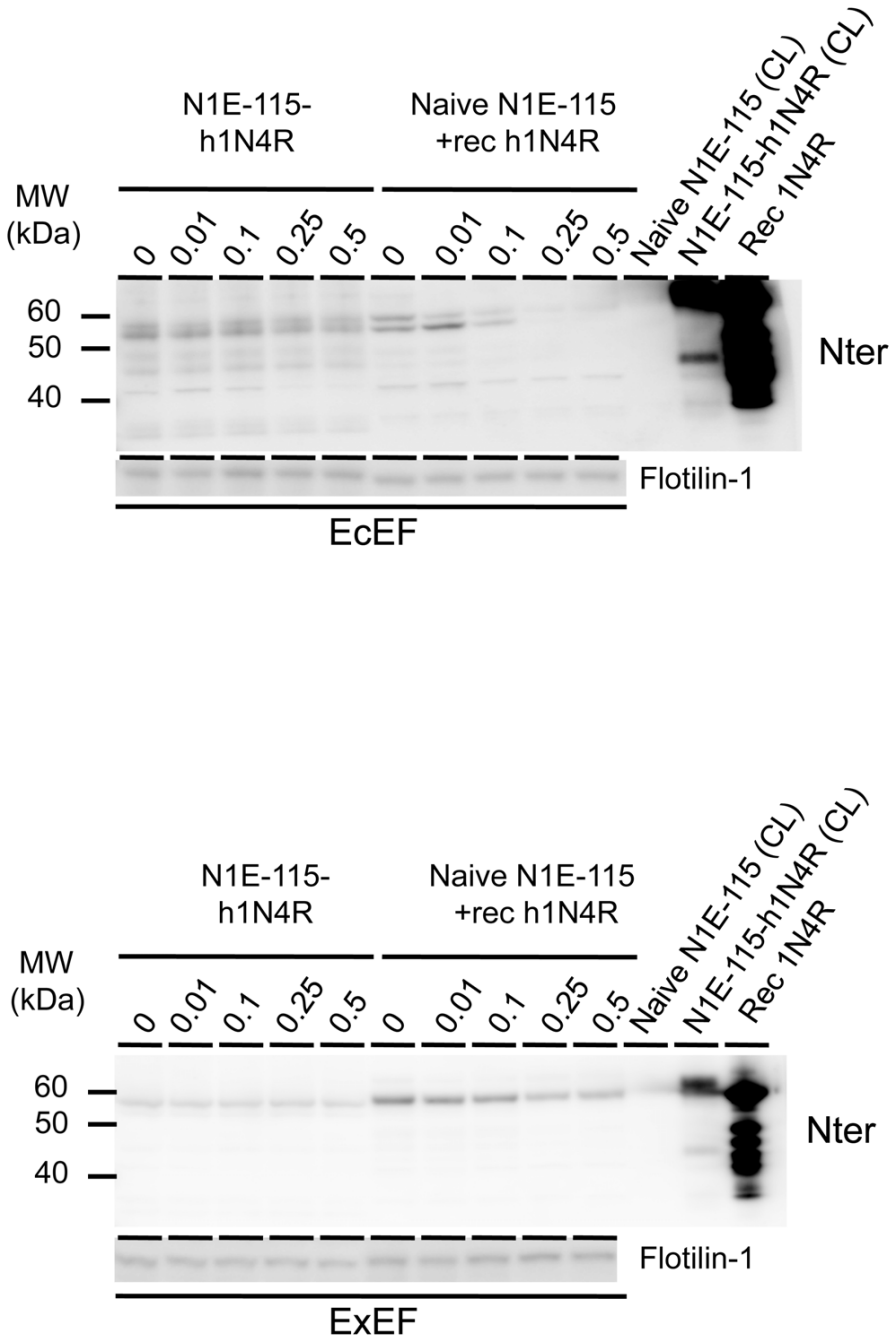
Tau is inside the vesicles. EcEF (upper panel) and ExEF (lower panel) obtained from stable N1E-115 overexpressing h1N4R cells (left part of the immunoblots) or from naive N1E-115 (right part of the immunoblots) were incubated with growing concentrations of NaCl (0.01 to 0.5M) before western blotting analyses using a total Tau N-ter antibody and a flotillin-1 antibody. EcEF and ExEF obtained from naive N1E-115 were previously incubated with recombinant h1N4R Tau. Cell lysates (CL) from both cell lines and recombinant h1N4R Tau were used as controls.

One may ask if Tau is really inside the vesicles and not stuck on vesicular surface. To demonstrate that Tau is intravesicular, ExEF and EcEF fractionated from N1E-115 cells over-expressing h1N4R were treated with increasing NaCl concentrations (from 0.01 to 0.5M) to remove Tau interacting with the extravesicular surface. Recombinant Tau (h1N4R) was added to extravesicular fractions from naive N1-E115 cells as a control to validate the effect of NaCl treatment. As expected, extravesicular recombinant Tau was removed from plain/naive vesicles whereas NaCl treatment did not affect Tau-immunoreactivity from EcEF/ExEF demonstrating that Tau is indeed inside vesicles or anchored to the internal vesicles membrane (**Fig. 5**).

In conclusion, the differential distribution of secreted Tau into particular fractions may be considered as a sensor of the physiological state of neurons. When this latter is deregulated, protein accumulation occurs; specific mechanisms of proteolysis, such as macroautophagy, are activated and result in Tau targeting to MVBs [46].

### Neurofibrillary degeneration related to WT Tau in the rat brain supports vesicular Tau secretion in ISF

**Figure 6:**
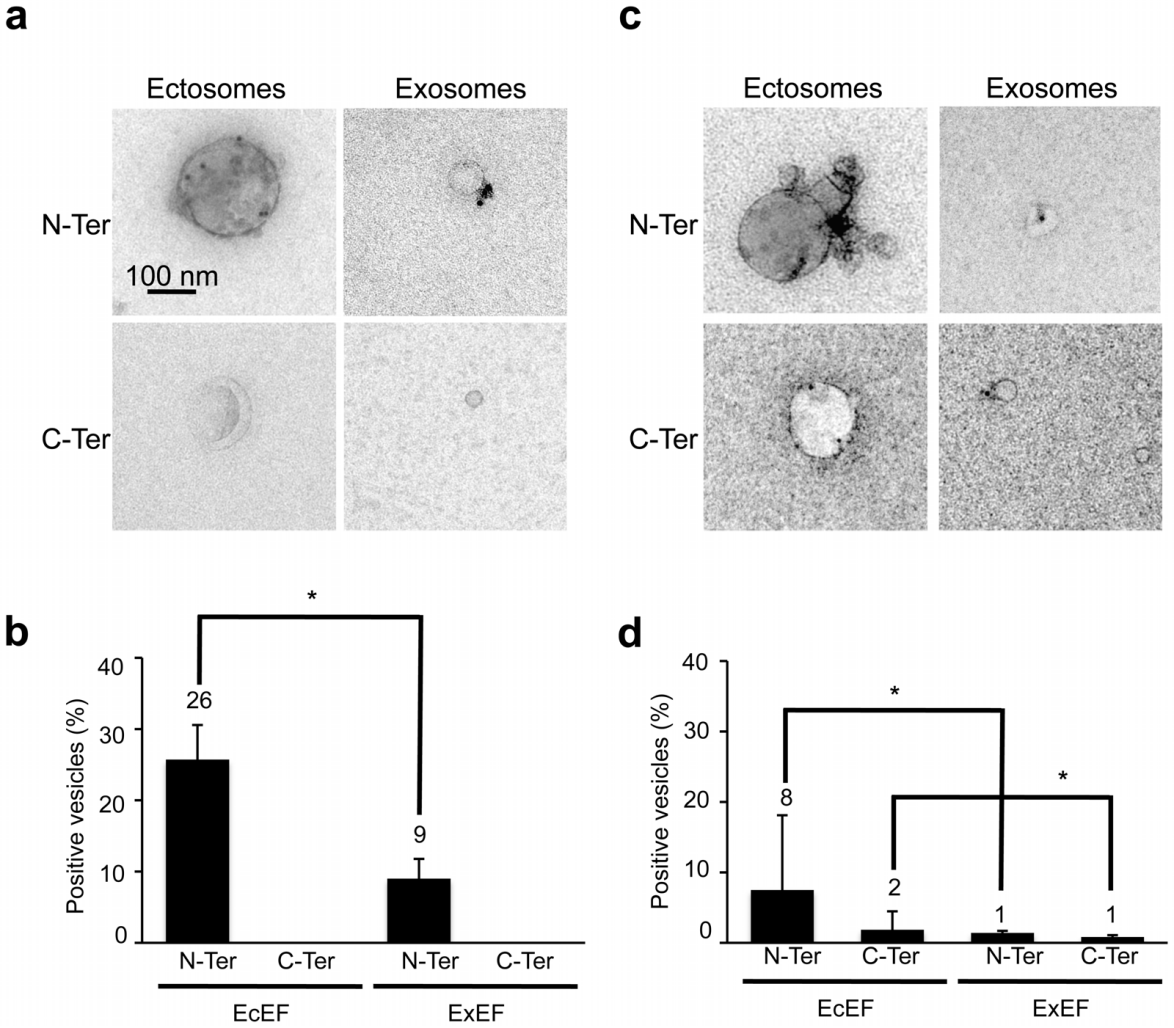
Neurofibrillary degeneration related to WT Tau in the rat brain supports vesicular Tau secretion in ISF. LVs encoding h1N4R were bilaterally injected into the CA1 layer of rat brains (n = 4). Five months later, ISF was recovered by the push-pull method. Naive control rats were also included in the assay (n = 2). EcEF and ExEF from LVs-injected rats (respectively **a, left panels** and **a, right panels**) or naive rats (respectively **c, left panels** and **c, right panels**) were fractionated and immunolabeled with N-Ter antibodies (LVs-injected rats: **a, upper panels**, naive rats: **c, upper panels**) or C-Ter antibodies (LVs-injected rats: **a, lower panels**, naive rats: **c, lower panels**). The scale bar is indicated on the figure. The association of Tau with vesicles was observed by EM using a 18 nm gold colloidal goat anti-rabbit antibody. A semi-quantitative analysis was performed to determine the percentage of ectosomes (size > 100 nm) and exosomes (30 nm < size <70 nm) immunopositive for Tau (LVs-injected rats: **b**, naive rats: **d**). For LVs-injected rats 4 independent experiments were performed; the total number of vesicles evaluated was 108, 110, 24 and 105 for C-Ter in ExEF, N-Ter in ExEF, C-Ter in EcEF and N-Ter in EcEF, respectively. For naive rats, 2 independent experiments were performed; the total number of vesicles evaluated was 254, 297, 35 and 27 for C-Ter in ExEF, N-Ter in ExEF, C-Ter in EcEF and N-Ter in EcEF, respectively. The results are expressed as the mean +/^_^ standard deviation and indicated at the top of the bar. The Chi^2^ test was used to compare the presence of Tau in ectosomes and exosomes: LVs-injected rats, N-Ter: Chi^2^ = 6.3 (*, p < 0.05); Naive rats, Nter: Chi^2^ = 10.9 (*, p < 0.05); Naive rats, Cter: Chi^2^ = 5.5 (*, p < 0.05).

We used our previously described rat model of progressive sporadic tauopathies to analyse Tau secretion [36], [41]. In this model, injection of LV-encoded h1N4R into the hippocampal formation mediates neurofibrillary degeneration. We wondered if this over-accumulation of WT Tau in neurons *in vivo* also results in secretion of Tau from neurons to the interstitial fluid (ISF). To address this question, we injected LV-h1N4R (n = 4 rats) in the hippocampal formation and performed push-pull experiments at the injection site at five months post-delivery. EcEF and ExEF were fractionated from these samples, and the presence of Tau was determined using EM (**Fig. 6a**). Semi-quantitative analyses showed that in ISF, Tau is secreted into vesicles, mainly in ectosomes (26%) but also to a slight extent in exosomes (9%) (**Fig. 6b**). We were unable to detect a signal using an antibody against the C-terminus of Tau, which shows that in this model of Tau overexpression, vesicular WT Tau species are mainly secreted without their C-terminus. We also performed push-pull experiments in naive rats (n = 2) to compare the level of Tau associated to vesicles in physiological conditions. These results gave the same conclusions generated by our *in vitro* experiments: when Tau is overexpressed, it is found associated to ectosomes but also exosomes whereas in physiological conditions Tau is only detected in ectosomes (**Fig. 6c and d**).

Our data confirm that Tau is secreted in the ISF as previously shown in WT and transgenic mice overexpressing a mutant form of Tau P301S [47]. To be relevant to sporadic tauopathies, which comprise most known tauopathies, we addressed the mechanism of human WT Tau secretion. Here, we provide evidences that when over-accumulated in neurons, WT Tau is released from neurons to the ISF at least partly through vesicular pathways (mainly ectosomes but also exosome vesicles).

The nature of the Tau species that are secreted is not yet known; however, the species type is mostly likely determined by a complex system involving Tau phosphorylation, Tau isoforms and Tau proteolysis. The proteolysis state of secreted Tau is controversial. Truncated forms of intracellular Tau, which lack the C-terminus, are detected in cell models [18], [48], [49] and tangles from autopsy-confirmed AD brains [48], [50], [51]. Similar truncated species are found in cell culture media obtained from cellular models or CSF-Tau from transgenic mice models [15], [52], in addition to human CSF [18], [50], [53]. However, full-length forms of secreted Tau are also found in cell culture media [13], [14], murine ISF [47] and human CSF [53]. By only evaluating the vesicular forms, we reconciled both previous observations, i.e., the presence of full-length secreted Tau in physiological conditions and truncated species in cases of overexpression. However, the role of proteolysis in Tau secretion is still unknown; proteolysis may facilitate Tau secretion, as observed for secretion of interleukin 1 [54]. When Tau is overexpressed, the appearance of proteolytic fragments lacking the C-terminus of Tau associated with ectosomes could also reflect the activation of caspases that precedes the formation of tangles in the transgenic mouse model [55], [56].

Because phosphorylation of Tau alters its association with the PM [57], it is not surprising that a dephosphorylated form of Tau would be more susceptible to secretion [14], [52], [58]-[60], thus leading to higher transfer of neuronal toxicity [61]. Our previous work indicated that secreted Tau is mainly dephosphorylated [41]. Dephosphorylated Tau species are actively secreted and are not derived from ghost tangles, but they are found in human CSF and used for diagnosis [39]. The nature of toxic species supporting the spreading of Tau pathology is still controversial and whereas some researchers argue that fibrils are the toxic species [62], others consider that soluble forms of Tau including oligomers, an intermediate form between monomers and fibrils are the seeding forms [63]. Moreover, the contribution of secreted Tau in the disease progression is unclear and one can speculate that a soluble form could rather be implicated in a physiological release of Tau [12], [13] whereas the fibrils forms could be the pathological species. Nevertheless, a recent study of Kayed's group demonstrated that passive immunization with Tau oligomer monoclonal antibody reverses tauopathy phenotypes [64]. In our hands, no fiber was seen by electron microscopy in extracellular vesicles from any experimental models and thus, soluble Tau species are likely to be present in these vesicles.

Another parameter, for which limited data are available, is how Tau isoforms specificity - resulting from alternative splicing of exons 2, 3, and 10 - may affect secretion. We performed our study using differentiated primary cultures and adult rat that both express the six Tau isoforms (data not shown and [36]). One study shows that Tau secretion is specifically inhibited by the presence of the exon 2 in transfected neuronal lines [65]. Nevertheless, the influence of exon 2 is still debated. A recent study indicates that the presence or absence of this exon has no influence on extracellular levels of Tau in SH-SY5Y cells [14]. In the present work, we demonstrated that the h1N4R is secreted indicating that the sequence encoded by exon 2 may not be crucial in secretion. However, knowing the differential microtubule binding property of Tau isoforms and their differential secretion in cell lines, additional studies are warranted to address these critical questions. Recently, differential subcellular localization of Tau isoforms in nucleus, cell bodies and dendrites has been reported [66]. Using isoform-specific antibodies Liu and collaborators show that there is a pronounced dendritic expression of the 1N and 2N isoforms. Tau amino-terminal domain (projection domain) plays a role in the interaction with the PM [10] and this interaction is dependent on phosphorylation [58]. Whether this is due to interactions with dendritic/synaptic proteins remains to be established. In addition, they show that the 1N Tau is enriched in the nucleus while 2N Tau is expressed mainly in cytoplasmic and axonal compartments.

To conclude, we demonstrated for the first time, *in vitro* and *in vivo* in a rat model of sporadic tauopathy, that Tau protein is present in the extracellular fluid in a new type of vesicles, i.e., ectosomes. Under basal conditions (rat neuronal primary cultures), in addition to major free forms, Tau is actively secreted through secretory pathways - rather in ectosomes than in exosomes. Extracellular vesicles have emerged as important mediators of intercellular communication, being involved in the transmission of biological signals between cells in both prokaryotes and higher eukaryotes to regulate a diverse range of biological processes but also in pathological process (For review see [67]). Moreover, it appears that cancer cell-derived extracellular vesicles containing many proteins, RNA and lipids participate to the cancer progression, which involves invasion, immune modulation, neovascularization, and metastasis (For review see [68]). Tau secretion from primary/iPS cells has been documented [12], [13], and this mechanism may be regulated by neuronal activity [12]. Here, we provide evidence that an ectosomal pathway at least partly mediates this secretion. These specific vesicles, directly emerging from the PM, enabled cytosolic Tau to be shuttled to the extracellular medium. Over-accumulation of intra-cellular Tau results in targeting to MVBs, leading to release in exosomes. This deregulation of Tau secretion could participate in the pathological spreading of Tau [69], as we also detected Tau in vesicles in our rat model of sporadic tauopathy.
